# Na^+^-Coupled Respiration and Reshaping of Extracellular Polysaccharide Layer Counteract Monensin-Induced Cation Permeability in *Prevotella bryantii* B_1_4

**DOI:** 10.3390/ijms221910202

**Published:** 2021-09-22

**Authors:** Andrej Trautmann, Lena Schleicher, Jana Pfirrmann, Christin Boldt, Julia Steuber, Jana Seifert

**Affiliations:** 1 HoLMiR-Hohenheim Center for Livestock Microbiome Research, University of Hohenheim, 70599 Stuttgart, Germany; andrej.trautmann@uni-hohenheim.de (A.T.); lena.schleicher@uni-hohenheim.de (L.S.); julia.steuber@uni-hohenheim.de (J.S.); 2 Institute of Animal Science, University of Hohenheim, 70599 Stuttgart, Germany; Jana.Pfirrmann@uni-hohenheim.de; 3 Institute of Biology, University of Hohenheim, 70599 Stuttgart, Germany; 4 Institute of Bioscience, TU Bergakademie Freiberg, 09599 Freiberg, Germany; Christin.Boldt@ioez.tu-freiberg.de

**Keywords:** monensin, *Prevotella bryantii* B_1_4, proteomics, Na^+^-translocating NADH:quinone oxidoreductase, extracellular polysaccharides, biofilms

## Abstract

Monensin is an ionophore for monovalent cations, which is frequently used to prevent ketosis and to enhance performance in dairy cows. Studies have shown the rumen bacteria *Prevotella bryantii* B_1_4 being less affected by monensin. The present study aimed to reveal more information about the respective molecular mechanisms in *P.* *bryantii*, as there is still a lack of knowledge about defense mechanisms against monensin. Cell growth experiments applying increasing concentrations of monensin and incubations up to 72 h were done. Harvested cells were used for label-free quantitative proteomics, enzyme activity measurements, quantification of intracellular sodium and extracellular glucose concentrations and fluorescence microscopy. Our findings confirmed an active cell growth and fermentation activity of *P.* *bryantii* B_1_4 despite monensin concentrations up to 60 µM. An elevated abundance and activity of the Na^+^-translocating NADH:quinone oxidoreductase counteracted sodium influx caused by monensin. Cell membranes and extracellular polysaccharides were highly influenced by monensin indicated by a reduced number of outer membrane proteins, an increased number of certain glucoside hydrolases and an elevated concentration of extracellular glucose. Thus, a reconstruction of extracellular polysaccharides in *P.* *bryantii* in response to monensin is proposed, which is expected to have a negative impact on the substrate binding capacities of this rumen bacterium.

## 1. Introduction

The prudent use of antibiotics in livestock farming is a crucial aspect for health of a dense stock. Monensin is a commonly used antibiotic in poultry farming and cattle fattening [1]. The application of monensin as a growth promotor in livestock farming became restricted to therapeutic interventions since 1996 in Denmark, 1999 in Switzerland and 2006 in the European Union [2,3,4]. An overuse of antibiotic growth promotors was regarded as concerning due to the formation of antibiotic resistance mechanisms. Therefore, utilization of monensin is restricted to the treatment of ruminal ketosis, acidosis and coccidiosis in dairy cows [5,6,7], and monensin supplementation to poultry feed was permitted again in 2020 by the European Union [8].

Monensin and its derivates were originally isolated from the soil bacteria *Streptomyces cinnamonensis*, which synthesizes the compound via the precursor butyrate and iso-butyrate [9,10,11]. A self-intoxication by monensin is prevented due to the anti-isobutyrate factor (AIB), which allows *S. cinnamonensis* to resist not only monensin, but also toxic concentrations of short-chain fatty acids (SCFAs) [12]. Several rumen microorganisms such as *Selenomonas ruminantium* and *Methanobacterium ruminantium* appeared to be insensitive towards monensin, while *Fibrobacter succinogenes*, *Bacteroidetes succinogenes*, *Prevotella ruminicola* and *Bacteroidetes ruminicola* showed a retarded growth in the presence of monensin [5,13]. Monensin is classified as an ionophore, which disrupts gradients of monovalent cations [14]. Multiple studies showed an increased cross-resistance of ruminal microorganisms, after exposure to sub-lethal concentrations of ionophores such as lasalocid, nigericin, valinomycin, tetranosin or monensin [13,14,15,16,17]. Callaway and Russell [14] summarized several resistance mechanisms: uncoupler translocase to transfer monensin away from its target location [18], change of membrane fatty acid composition to prevent monensin binding [19], reduction of porin size for passage inhibition [5,16] and formation of extracellular polysaccharides (EPS) to prevent monensin binding to its target [20]. Monensin cell binding can also be reduced by TRIS-EDTA washing, as it was shown for mixed ruminal bacteria [21].

In comparison to other *Prevotella* species, *P. bryantii* B_1_4 was less affected by high concentrations of monensin (20 µM) and showed a slightly reduced cell density [22]. Especially in the ruminal ecosystem, *P. bryantii* B_1_4 seemed to be more resilient towards monensin exposition [23]. Higher concentrations of monensin (up to 60 µM) were also tolerated by *P. ruminicola* strain 23 and strain GA33 [15].

In the present study, monensin adaptation mechanisms in *P. bryantii* based on proteomic, enzymatic and microscopic results are shown. The study elucidates upcoming general resistance strategies in bacteria towards ionophore antibiotics involving cell surface modifications promoting for biofilm formation.

## 2. Results

### 2.1. Inhibition of Prevotella Bryantii B*_1_4* Growth by Increasing Monensin Concentrations

**Figure 1:**
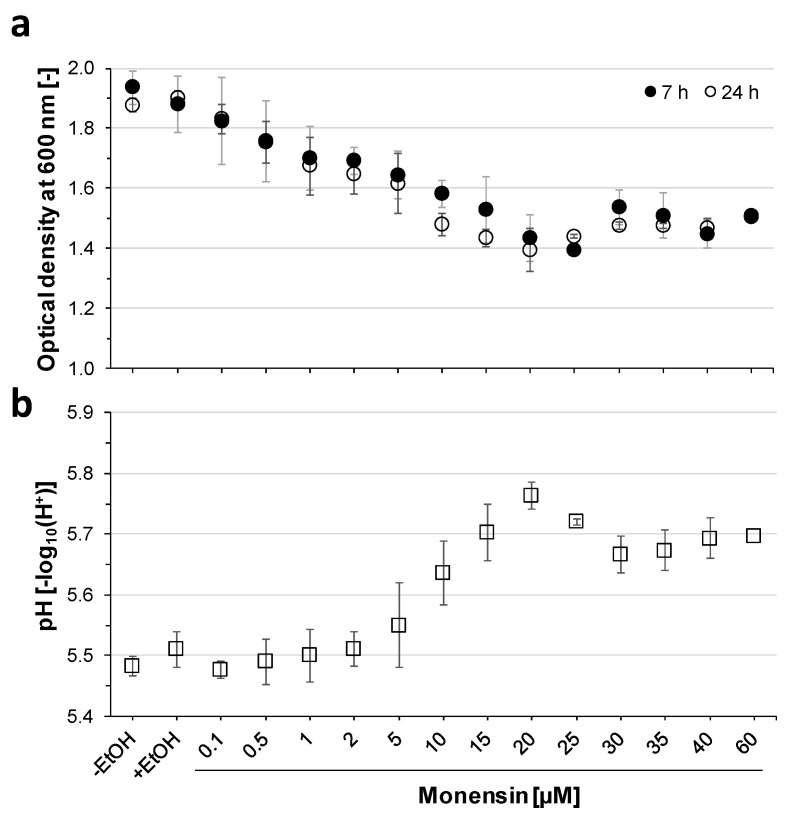
Optical density and pH values of *P. bryantii* B_1_4 using a range of monensin concentrations in Hungate tube cultures. (**a**) Mean optical density (OD) after 7 h (black) and 24 h (white) of incubation are given with error bars for standard deviation (*n* = 3). No significant difference was observed between 7 and 24 h (ANOVA: *p* = 0.486). (**b**) The pH average after 24 h (white squares) of incubation are given with error bars for standard deviation (*n* = 3). Two controls without monensin excluded or included (−/+) EtOH. Significances were proven by Fisher LSD-test (*p* < 0.01).

Dose-dependent effects of monensin on *P. bryantii* growth were tested using 15 concentrations in a range between 0.1 to 60 µM. Optical density (OD) was monitored every 30 min for 7 h. An analysis of variance showed no significant differences between the ODs at 7 and 24 h of incubation (*p* = 0.486, Figure 1a). OD decreased gradually from OD_24h_ = 1.90 at 0 µM to the lowest OD at 20 µM monensin (OD_24h_ = 1.39). The minimal inhibitory concentration (MIC) defined by significance in OD was shown at 1 µM (*p* < 0.01). The strongest growth inhibition was seen with 20 µM, whereas the subsequent monensin treatments (30, 35 and 60 µM) showed a slight increase of the OD, indicating a degressive dose-dependent relationship (Figure 1a). A mirrored development was seen for pH values showing the highest pH at 20 µM (pH_24h_ = 5.76; *p* < 0.01; Figure 1b). The lowest pH, meaning the highest acid production, was observed under 0.1 µM monensin (pH_24h_ = 5.48). Treatments above 10 µM monensin revealed a lag phase of 3.5–4 h, while without monensin, a maximum of 1 h lag-phase was determined. The slowest growth rate was found at 20 µM monensin (Figure S1). Control cultures incubated with EtOH (1.3% (*v*/*v*)) showed no effect on final OD, whereas pH was higher with EtOH as without (*p* = 0.047).

**Figure 2:**
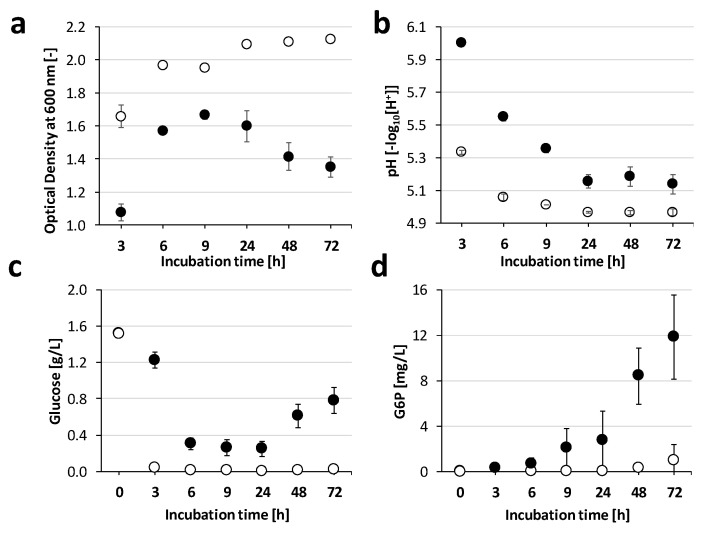
Growth and fermentation in the presence of monensin triggers intracellular sodium efflux in *P. bryantii* B_1_4. (○ 0 µM and • 20 µM). Parameters were obtained from 100 mL cultivations after 9 h growth under monensin concentrations (0, 10, 20 and 50 µM). The average of six biological replicates was displayed for every measurement and condition. Error bars indicate standard deviation if not stated separately. (**a**) Optical density. (**b**) pH, adjusted pH of 6.8 is displayed for the non-inoculated pure medium. (**c**) Intracellular Na^+^ concentration analyzed by ICP-MS and standardized by the protein concentration. The treatment without monensin was set as reference (100%) and error bars indicate standard error mean (SEM). (**d**) Relative D-glucose concentration in supernatant, which is in pure medium below the supplemented 2 g/L due to the Maillard reaction during autoclaving.

Four concentrations (0, 10, 20 and 50 µM) were selected for cultivation in up-scaled volumes (v = 103 mL). Six biological replicates per monensin concentration were harvested after 9 h of incubation and OD, pH, intracellular sodium, glucose in culture supernatant and proteome of *P. bryantii* cells were analyzed. A lowered OD was observed at monensin concentrations of 20 µM and 50 µM, as compared to the Hungate tube cultures (OD of 1.4 vs. 1.0 in 20 µM monensin treatments, Figure 2a). The pH changed significantly between the treatments, except for 20 and 50 µM (Figure 2b).

Intracellular sodium concentrations were monitored to determine the effect of the ionophore monensin on *P. bryantii* B_1_4 cells. Mean intracellular Na^+^ concentration of 128 µg sodium per milligram protein was found in the control (without monensin) and set as reference (Figure 2c). In the presence of 10 µM monensin, intracellular Na^+^ was significantly reduced (*p* < 0.01) to 33 µg/mg protein and increased with rising monensin concentration to 105 µg/mg protein (Figure 2c).

Glucose consumption was measured to determine the effect of monensin towards sugar uptake and fermentation activities. Without monensin almost all glucose was consumed, which resulted in a leftover of 0.06 g/L after 9 h of incubation. A residual amount of glucose in the medium was found for 10 µM monensin (0.2 g/L), while at higher monensin levels (20 and 50 µM), about 0.8 and 0.9 g/L of glucose remained in supernatant (*p* < 0.01, Figure 2d).

### 2.2. Growth Adaptations towards Monensin over Time

**Figure 3:**
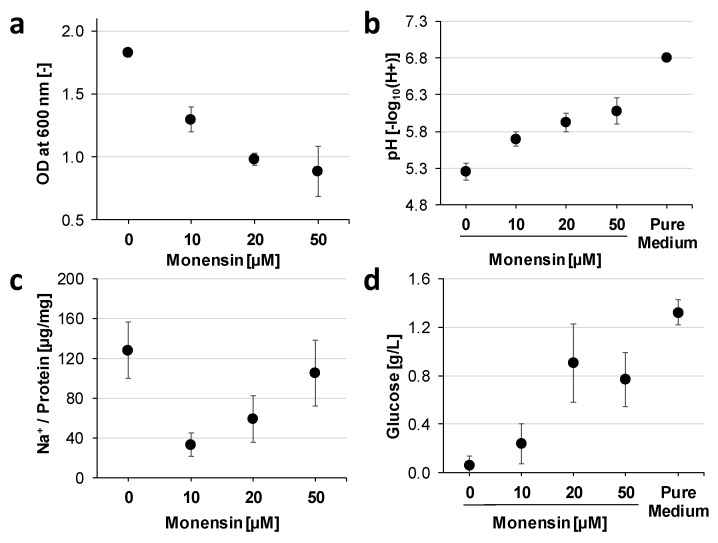
Glucose and glucose-6-phosphate in supernatant are displayed over time in two monensin conditions. Small letters indicate least significant difference (LSD) between time points for monensin group with *p*-values indicated in brackets. Measurements for 0 and 20 µM were significantly different (*p* < 0.01). Measuring at 0 h was performed with non-inoculated medium. All measurements display the average as a dot and error bars as standard deviation (SD). (**a**) Optical density at 600 nm wavelength (*p* < 0.01). (**b**) pH of cultivation media (*p* < 0.01). (**c**) Extracellular glucose only with LSD-test for 20 µM (*p* < 0.01). (**d**) Extracellular glucose-6-phosphate (*G*6*P*) only with LSD-test for 20 µM (*p* < 0.05).

A prolonged incubation (up to 72 h) with 20 µM monensin showed a significantly decreased cell density (*p* < 0.001) at all-time points (Figure 3a), while OD without monensin remained stationary and pH dropped faster and achieved a lower pH as with monensin (Figure 3b). In absence of monensin, glucose was already consumed after 3 h, while with 20 µM monensin, glucose concentration was stable from 6 to 24 h on a low level and increased significantly at 48 and 72 h of incubation (*p* < 0.01, Figure 3c). Glucose-6-phosphate was quantified concomitantly with glucose, showing a significant increase starting at 48 h (*p* < 0.01, Figure 3d).

### 2.3. Dose Dependent Modulation of the Proteome of Prevotella bryantii B*_1_4* by Monensin

**Figure 4:**
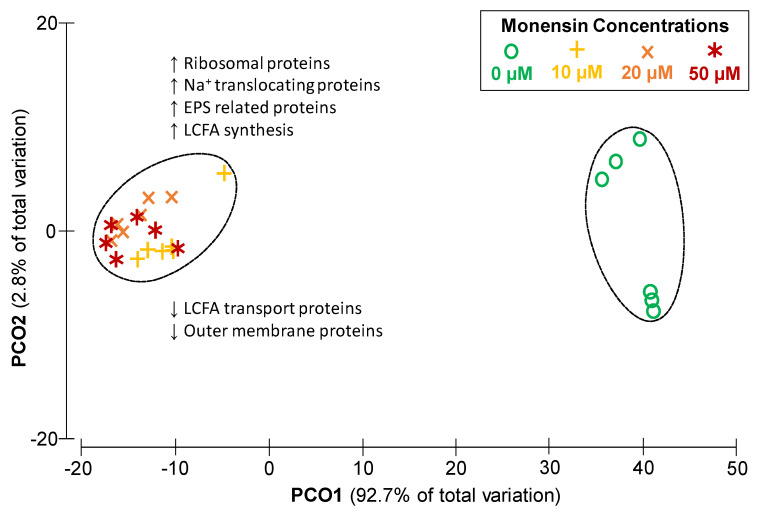
Changes of *P. bryantii* proteomes at different monensin conditions. Each condition consisted of six replicates (*n* = 6). Principal component analysis with 1686 label-free quantified proteins resulted in two clusters of 80% similarity dividing in with (**left**) and without monensin (**right**). Monensin concentrations are depicted in green (0 µM), yellow (10 µM), orange (20 µM), and red (50 µM). Arrows with functional assignation indicate a high (↑) or low (↓) abundance of mentioned functional protein groups.

*P. bryantii* B_1_4 cultures were exposed to 0, 10, 20 and 50 µM monensin, from which proteins were extracted after 9 h of cultivation. A total of 1686 proteins were identified and quantified in at least one sample (Data S1). The number of label-free quantified proteins ranged from 985 to 1349 proteins per sample (Table S1). All cultivation conditions shared 905 proteins (Figure S2), while all samples shared 742 proteins. The principal component (PCO) analysis (Figure 4) showed a total variation of 95.5% between samples with and without monensin. Both clusters showed a similarity of 80% within each other, while 20% of proteins were shared with a similar expression pattern (fold-change < 1.5). Minor differences in protein abundances among monensin treated cells were observed in a low fraction of proteins (2%; 35 proteins), whereas most proteins (64.7%; 1091 proteins) revealed a fold-change difference of more than two.

Changes of functional proteins showed a major shift in clusters of orthologous groups (COGs) during monensin supplementation (Table S2). Proteins belonging to the COGs of translation, ribosomal structures and biogenesis (J), post-translational modification, protein turnover and chaperones (O), transcription (K), energy production (C) and intracellular trafficking, secretion and vesicular transport (U) were significantly elevated in abundance (≥1%) with monensin. COGs responsible for cell wall-, membrane- and envelope biogenesis (M), as well as ion transport and metabolism (P) and a vast number of proteins with unknown functions (S, S!), were significantly depleted in monensin treated cells.

The number of identifications and abundance of outer membrane proteins decreased in monensin treated cells (Table S3). Proteins involved in iron and fatty acid transport as well as defense mechanisms were less abundant in monensin cultures. Several miscellaneous transporters targeting cations (Na^+^, K^+^, Ca^2+^ and Zn^2+^), peptides, amino acids, sugars and phosphate were more abundant in monensin treatments (Table S4). Despite enhanced expression of iron related transporters at 0 µM monensin, ferrous iron transport protein B (feoB, D8DT71) was increased around 15–17 fold in the presence of monensin (Table S4). Additionally, import related proteins (TonB, RagB/SusD, IPT/TIG domain containing) highly correlated (R ≥ 0.7) with intracellular Na^+^ concentration (Table S5).

A 5-fold higher abundance was seen for the phospholipid/cholesterol transport protein (A0A1H8YR75) in monensin cultures. Abundance of most detected ribosomal proteins increased under monensin supplementation, except 50S subunit ribosomal protein L29 decreased, which was positively correlating with OD and pH (R² = 0.78 and R² = 0.77).

**Figure 5:**
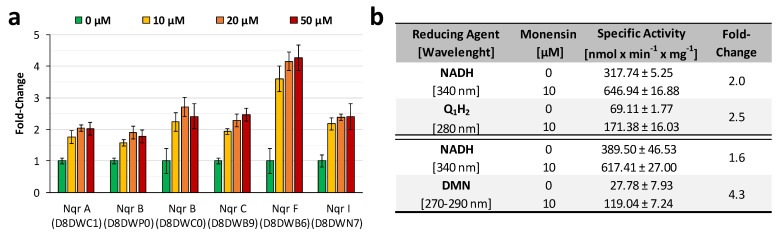
Na^+^-translocating NADH:quinone oxidoreductase (NQR) abundance and activity under presence or absence of monensin. (**a**) Bar plot with mean abundance (*n* = 6) of NQR subunits including their standard deviation represented as error bars. The Uniprot ID is given in brackets. (**b**) Mean specific activity (*n* = 3) and standard deviation of NQR. Oxidation NADH, and formation of ubiquinone (Q_1_H_2_) and menaquinole (DMNH_2_) were followed with membranes of *P. bryantii* B_1_4.

Special emphasis was given on sodium transport proteins like the Na^+^/H^+^ antiporter NhaD (D8DYG5) and the phosphate Na^+^-symporter (A0A1H9BJD7), which were increased concomitantly among all monensin doses (Table S4). The sodium/glucose cotransporter (D8DYQ7) was quantified in all treatments and increased gradually with the monensin concentration (Table S4). Subunits of Na^+^-translocating NADH:quinone oxidoreductase (NQR), which are involved in anaerobic respiration and quantified at all culture conditions were analyzed under increasing monensin concentrations (Figure 5a). Similar to Na^+^ transporting proteins, NQR subunits were also differently abundant and ranging from 1.5 to 4-fold compared to proteins without monensin, which were set to a fold-change of one. In total, NQR proteins were more abundant during monensin supplementation and increased with prolonged incubation time (Table S6). Observed differences of the NQR abundance in *P. bryantii* cells were confirmed by measurements of NADH oxidation and 2,3-dimethyl-1,4-naphthoquinone reduction activity in isolated membranes of *P. bryantii* grown without or with 10 µM monensin (Figure 5b). The fold-change in activity was set for 0 µM monensin to one and showed a 2–4.3-fold higher activity in 10 µM monensin cultures.

Twenty-four uncharacterized proteins of *P. bryantii* B_1_4 exceeded a fold-change greater than five in monensin cultures. Blast search of five of those resulted in a score of 90 and functional assignments were proposed (Table S7). Protein D8DVZ9 revealed in Blast search the highest similarity to the YghO protein, which is known to act as an N-acetyltransferase.

### 2.4. Protein Modification in Monensin Cultures over Time

**Figure 6:**
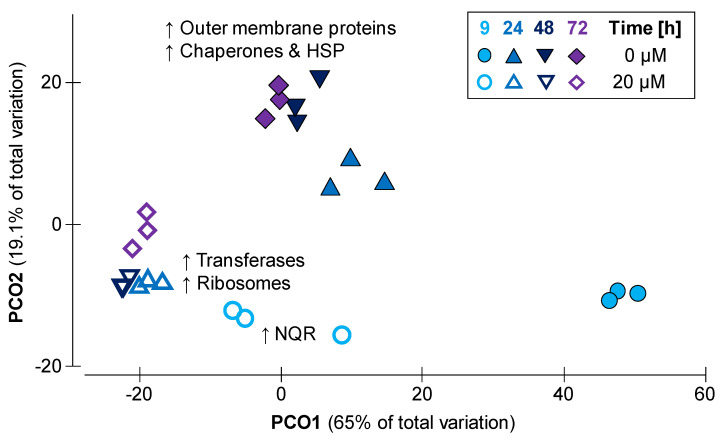
Changes of *P. bryantii* proteomes at 0 and 20 µM monensin after 9, 24, 48 and 72 h of incubation (*n* = 3 per time point and treatment). Data underwent a standardization by total, followed by resemblance matrix formation via Bray Curtis similarity.

*Prevotella bryantii* B_1_4 was cultivated with 0 and 20 µM monensin for 72 h, and samples were taken at four different time points. Whole data sets showed a variation of 84.1% according to time and treatment (Figure 6). Samples grouped mainly according to presence and absence of monensin and shifted by time within their grouping along PCO1. Peptide and protein counts are listed in Table S8. Including all time points, 625 proteins for control and 942 proteins for monensin conditions were quantified (Data S2, Figure S3). After 9 h, 178 proteins (14%) appeared in the following time points of the control group. The monensin exposed proteome showed 222 proteins (18%), shared by all time points except 72 h. Both conditions showed a small but dominant fraction (9–11%) of proteins, which appeared only at 9 h. Relative abundance of COG classes revealed a significant difference between the control and the monensin-supplemented cultivation for the majority of proteins (Table S9). Samples without monensin showed elevated abundances of outer membrane proteins and proteins of post-translational modification (Table S9). Proteins of the monensin cultures shifted in a minor scale as the control and had increased abundances of transferases, ribosomes as well as a higher level of NQR at the beginning of the cultivation. COGs that were more abundant in monensin-cultivated cells belonged mainly to translation, ribosomal structure and biogenesis (J), energy production and conversion (C) and carbohydrate transport and metabolism (G). Most abundant COGs in the control belonged to inorganic ion transport and metabolism (P), function unknown (S and S!), cell/membrane/envelope biogenesis (M) and post-translational modification, protein turnover and chaperones (O). Most proteomic differences between time points appeared between the late exponential phase (9 h) and the residual time points (Figure 6).

Proteins belonging to the COG classes of the energy production and conversion (C) as well as the carbohydrate transport and metabolism (G) were closer investigated due to the observed increase of extracellular glucose in monensin supplemented cells after 24 h (Figure 3c). No clear elevation of proteins connected to gluconeogenesis were observed, but an increase of glucosidases responsible for conversion of maltose, cellobiose, dextrin, cellodextrin, starch, and β-glycosides to glucose was identified (Table S10). Seventy-two different carbohydrate-active enzyme families (CAZymes) showed a predominance of GH13 and GH2 in monensin-supplemented cells, while families PL1 and GH43 were predominant in the control (Table S11). Family GH2 was equally distributed, while the distribution of proteins belonging to other CAZyme families could not be analyzed since they were underrepresented (proteins < 5).

### 2.5. Altered EPS Structure in Monensin Cultivations

**Figure 7:**
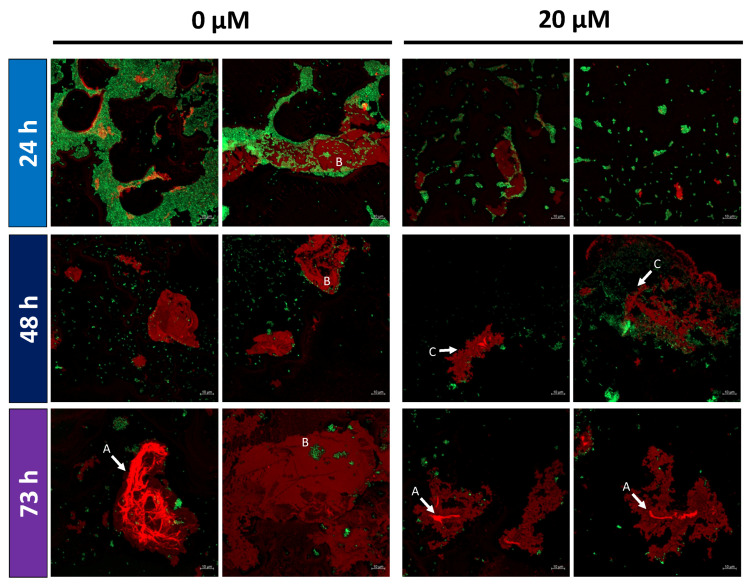
Fluorescence microscopy of *Prevotella bryantii* B_1_4 cells at 24, 48 and 73 h of cultivation with 0 and 20 µM monensin. Fluorescence stain of EPS (red, Alexa Fluor 594) and DNA (green, SYTO 9). Each condition is presented in duplicates. Lower right corner indicates a size reference of 10 µM. Capital letters point at dense EPS regions (**A**), cells embedded in EPS (**B**) and at fringed edges of EPS layer (**C**).

*Prevotella bryantii* B_1_4 cells were cultured without and with (20 µM) monensin for 24, 48 and 73 h, harvested, and processed for confocal imaging to analyze the effect of monensin on EPS structure. Double staining of EPS and nucleic acid (cells) confirmed structural changes in EPS under monensin supplementation (Figure 7). Compact and linear EPS structures were found in monensin cultivations, while EPS from control conditions appeared to be more branched and complex, especially seen in EPS dense regions (Figure 7A). In absence of monensin, cells were mostly embedded into the EPS, while in monensin cultures cells were dissolved out of the EPS (Figure 7B). After 48 h, fringed edges of EPS were observed with monensin-exposed cells, whereas EPS edges from untreated cells were blunt and smooth (Figure 7C). Additionally, a decelerated development of EPS was seen with monensin supplementation over time (Figure 7).

## 3. Discussion

### 3.1. Degressive Dose–Response to Monensin

Monitoring OD and pH in the first seven hours of monensin exposure revealed a degressive dose–response relationship and showed that a monensin concentration of 20 µM is the most effective for the inhibition of growth and fermentation activity in *P. bryantii* B_1_4. Callaway and Russell reported only a weak growth inhibition for monensin concentrations up to 20 µM [22]. For *Bacteroides ruminicola* (reassigned as *Prevotella ruminicola* GA33) a linear inhibition and slower growth was shown up to 40 µM monensin, but with less tested concentrations [13]. An explanation for the newly observed stagnation of the antibiotic effect beyond 20 µM monensin might be the maximum capacity of monensin accumulation into the membrane because of its lipophilic property [22]. Another impact on cell surface composition and cell division is caused by the murein layer deconstruction, which has to be performed in the area of the septal ring [24]. Increased abundance of cell division proteins (Clp, Fts) in monensin cultures (Table S12) with prolonged incubation seemed to compensate slower growth rate and lower OD.

### 3.2. Cation Permeability Affects Proteome and Metabolism

Growth inhibition caused by monensin is primarily explained by the disruption of the ion gradient for monovalent cations (K^+^, Na^+^, H^+^), which is caused by an increased cation permeability [14]. Considering the increased membrane permeability for protons, the intracellular pH is probably acidified with progressing fermentation [25,26]. Therefore, inhibition of cytoplasmic metabolism is presumed due to intracellular acidification, which is unfavorable for enzymatic activities. Since fermentation was therefore stopped after 24 h of monensin cultivation, glucose remained in the supernatant. Inhibition of glucose transport can be rejected, as sodium/glucose cotransporter (D8DYQ7) was even almost 3-fold elevated in monensin cultures on proteome level.

In a previous study, a decrease of 85% cytoplasmic K^+^ was described when 10 µM monensin was applied [22]. A similar finding was seen in the current study for Na^+^, suggesting an enforced Na^+^ efflux by elevated abundance of NQR and other Na^+^-translocating proteins. Since *P. bryantii* possesses only genes encoding for the menaquinone (DMN) synthesis, but not for ubiquinone (Q1) synthesis, the fold-change of NQR activity with DMN appears closer to in vivo conditions. The activity fold-change of NQR with DMN was similar to fold-change of the protein abundances. NQR activity and abundance did not increase gradually along the gradient of monensin concentrations, but in a switch-like manner, as seen in the quantitative proteome analysis. This observation points out that the active export of Na^+^ was a countermeasure with respect to the uncontrolled cation permeability caused by monensin. A high correlation (R ≥ 0.7) of Na^+^ concentrations with the abundances of certain outer membrane proteins might also be used as an indicator for the regulation of protein synthesis by the level of sodium in the cells. The Na^+^/H^+^ antiporter NhaD (D8DYG5) and cation translocating ATPases in *E. coli* and *Enterococcus* showed similar effects [27,28].

### 3.3. Monensin Triggers Extracellular Polysaccharide Degradation by P. bryantii with a Concomitant Release of Glucose

Glucose consumption was diminished with increasing monensin concentrations, while further investigation also showed an increase of glucose-6-phosphate, which is most likely derived from cell lysates. However, increasing glucose concentrations at 48 h and 72 h were unlikely to be formed in the gluconeogenesis pathway, as proteins responsible for the gluconeogenesis such as fructose bisphosphatase (EC 3.1.3.11) were not detectable in the peptide data. Morehead and Dawson [15] described a complete glucose consumption within 36 h for *Prevotella ruminicola* strains under 14 µM monensin, while remnants of glucose were found in the current approach. Furthermore, increased glucose concentration in the culture supernatant at 48 and 72 h can be explained by the degradation of EPS facilitated through glucosidases and the inhibited glucose uptake mechanism with monensin supplementation. Glucosidase assignments were done based on KEGG orthology (KO), enzyme classification (EC) and CAZyme classification suggesting hydrolytic capacities of *P. bryantii* to convert various poly- and monosaccharides (cellobiose, maltose, dextrin, starch, glycogen) into glucose (Table S13). A β-glucosidase (A0A1H9IED8), with the ability to hydrolyze cellobiose, increased equally in protein abundance to the extracellular glucose in monensin cultures. This possible correlation and influence of the β-glucosidase was supported by supplementary experiments where glucose concentrations were enhanced in the presence of cellobiose and monensin in the cultures (Figure S4). The α-glucosidase (D8DXY7), able to depolymerize non-supplemented starch and dextrin, showed a strong increase in abundance over time in monensin cultures. The corresponding family GH2 uses galactose and mannose as potential substrates, which can be found as main components in EPS of *P. bryantii* [29]. Aside from the mentioned glucosidases, a vast number of transferases, mostly for sugars, as well as the newly assigned protein N-acetyltransferase (YghO, D8DVZ9), were elevated in monensin cultures and are due to their functionality most likely connected to EPS conversion.

Glucose is used in EPS formation [30], and EPS may serve as a sacrificial layer against ionophore attachment on the cell membrane [20]. In the presented experiments, differences in stickiness of monensin-treated versus untreated cells during centrifugation and cell pellet suspension indicated an altered bacterial surface. This was also seen on the proteome level as outer membrane proteins were depleted in abundance. Previous studies described the impact of antibiotics on the phospholipid bilayer, EPS hydrophobicity and lipopolysaccharides in other microorganisms [17,31,32,33,34]. The cell surface and membrane was highly influenced by monensin as outer membrane proteins and a vast number of proteins belonging to COG class M (cell wall/membrane/envelope biogenesis) were quantitatively changed. The Do/DeqQ family serine protease (D8DWJ3), highly abundant in monensin growing cells, decreased during the prolonged incubation time and had therefore a less suppressive effect on EPS formation [35]. Biofilm formation in *A. baumannii* was accompanied by elevated outer membrane proteins and proteins of the histidine metabolism [36]. A similar observation was made for the histidine pathway (Figure S5) in the present experiment, but the opposite was found for outer membrane proteins.

Based on the present findings, the detachment and reconstruction of EPS in *P. bryantii* B_1_4 cells during monensin exposure is backed up by the following evidences:

1. An increased amount of EPS degrading enzymes such as glucosidases or COG M class proteins;
2. Elevated amounts of extracellular glucose;
3. Zones with EPS depletions in *P. bryantii* cell clusters.

These arguments point towards a partial EPS deconstruction in cells treated with monensin. This is also in line with the two-layer EPS model explained by Nielsen and Jahn [37], describing a dense and closely cell-attached inner layer, and a loosely bound and soluble outer EPS layer. Fluorescence microscopy showed loosely detached EPS layers with signs of decomposition in monensin exposed cells. Stability of EPS in these cells seemed also diminished while handling cell pellets and microscopic slide preparation in comparison to cells grown without monensin. The two-layer EPS theory would explain the findings of Chow et al. [21], who showed almost no binding of isotope labeled monensin in *P. bryantii* B_1_4 cells or vesicles. Extensive composition analysis of the outer EPS layer could provide insights about quality and amount of EPS as well as potential monensin and its binding partners such as cations, which are also favorably bound to EPS [38]. This could be done in future experiments using anaerobic flow-cell cultivation techniques.

A degressive dose–response relationship was observed for fermentation and optical density towards incrementing monensin concentration. Protein adaptations to monensin operated in a switch-like manner similarly to the intracellular Na^+^ efflux, which was most likely regulated via elevated NQR activity and other Na^+^ translocating proteins. Monensin was shown to have an impact on the outer cell membrane and the degradation of extracellular polysaccharides. An elevated abundance of certain glucoside hydrolases can be correlated to an increase of extracellular glucose. Thus, a detachment and partial degradation of EPS charged with monensin by *P. bryantii* is likely and can be described as a kind of protection or resistance mechanism. This should be evaluated in respect to the strain and its accompanied rumen community members, which need intact cell surfaces to bind and hydrolyze available substrates such as fibers.

## 4. Materials and Methods

### 4.1. Cultivation

*Prevotella bryantii* B_1_4 (DSM 11371) was cultivated under anaerobic conditions at 39 °C and a starting pH of 6.8 ± 0.05. Medium composition was based on the M2-B medium from Trautmann et al. [39] using glucose, maltose and cellobiose, instead of only glucose. Additionally, 1.4% (*v*/*v*) of a vitamin mix and monensin solution were injected into the Hungate tube via sterile filtration (pore size 0.2 µm) resulting in a total volume of 7.5 mL. A 100 µM monensin stock solution was prepared by solving monensin sodium salt (purity ≥ 90% Alfa Aesar, Haverhill, MA, USA) in pure EtOH (purity = 100%; Merck, Darmstadt, Germany). Cells grown in M2-B without monensin or EtOH supplementation and a final optical density (OD) of at least 1.8 were used for inoculation (4–10% *v*/*v*, depending on the used culture volume). Effects of monensin on growth, EPS formation and fermentation parameters were investigated by using Hungate tube cultures (7.5 mL final volume). Cultivation flasks (103 mL final volume) were used additionally for sodium determination and proteomics. Cultivation in serum bottles (1 L final volume) was performed for cell enrichment required for the enzymatic assays.

### 4.2. Growth in Presence of Monensin

A broad range of monensin concentrations (0, 0.1, 0.5, 1, 2, 5, 10, 15, 20, 25, 30, 35, 40 and 60 µM) was used to determine the effect of monensin on growth parameters (OD, pH) and to define the minimal inhibitory concentration (MIC) by using the first significant difference in optical density compared to the control. EtOH was added to control cultures with equal volumes as used with monensin in EtOH. Hungate tube cultures were incubated for 24 h in triplicates. OD at 600 nm was measured every 30 min within the first 7 h with the help of a densitometer (Bio Genesys^TM^ 10, Thermo Fisher Scientific, Darmstadt, Germany). After 24h, final OD and pH (pH meter, FE20, Mettler Toledo, Columbus, OH, USA) were determined.

### 4.3. Determination of Intracellular Sodium Content

*P. bryantii* B_1_4 was grown in anaerobic cultivation flasks (103 mL) with 0, 10, 20 and 50 µM monensin for 9 h (six replicates each). To the control (0 µM monensin), the respective volume of EtOH was added. OD and pH were measured by transferring four milliliters of the batch culture into a Hungate tube. Both measurements were conducted as described before. After 9 h, supernatant and cells were separated by centrifugation for 10 min at 8000× *g* and 4 °C. Cell pellets were stored at −80 °C.

Cell preparation and sodium measurements were based on previous studies [40,41]. Cell pellets were suspended in 30 mL of 100 mM Tris/HCl (pH 7.3) and adjusted to OD_600_ = 30. Cells were centrifuged (10 min; 8000× *g*; 4 °C), disrupted with 3 mL of 5% (*w*/*v*) trichloroacetic acid (TCA) by boiling for 20 min at 95 °C. The cell extract was centrifuged (20 min; 10,000× *g*; 4 °C) and the supernatant, containing the intracellular sodium, was stored at 4 °C. Cell debris were resuspended in 2 mL ddH_2_O, and protein content was determined with the Bradford assay [42].

Intracellular sodium concentrations of all six replicates were quantified by inductively coupled plasma mass spectrometry (ICP-MS). An aliquot (1 mL) of the sodium containing supernatant was diluted by adding 3 mL ddH_2_O and analyzed in the NexION device (Perkin Elmer, Waltham, MA, USA). The quantification was conducted as described in the literature for sodium analysis [43]. The amount of sodium was standardized by the amount of protein, determined in the suspended cell debris. K^+^ concentrations were below the limit of quantification by ICP-MS.

### 4.4. Determination of D-Glucose and Glucose-*6*-Phosphate in Cell Cultures

D-glucose and glucose-6-phosphate (*G*6*P*) concentrations were determined in supernatants from cell cultures using the specific conversion with hexokinase (Roche, Basel, Switzerland) and *G*6*P* dehydrogenase (Roche, Basel, Switzerland) coupled to the conversion of NAD^+^ to NADH in enzymatic assays. NADH concentrations reflecting sugar concentrations were followed at 340 nm [44]. The reaction mixture consisted of 100 µL diluted sample (1:10), 865 µL of Tris HCl (380 mM) with MgSO_4_ (6.4 mM) at pH 7.5, 20 µL of 100 mM NAD^+^ (Roth, Karlsruhe, Germany) and 10 µL of 100 mM ATP (Roth, Karlsruhe, Germany). Absorption at 340 nm (*E*1) was measured before adding 3 µL of *G*6*P* dehydrogenase (1000 U/mL) and incubation for 15 min at room temperature. After incubation, second measurement (*E*2) was performed with subsequent addition of 3 µL hexokinase (1500 U/mL) and further incubation as described before. Final absorption was determined after a stable absorption value was attained (*E*3). The parameters of sample volume (*v* = 0.1 mL), total volume (*V* = 1 mL), molecular weight of glucose (*Mw* = 180.16 g/mol) and *G*6*P* (*Mw* = 260.14 g/mol), cuvette thickness (*d* = 1 cm), extinction coefficient for NADH at 340 nm (*ε*_340_ = 6230 L × cm^−1^ × mol^−1^) the absorption difference for glucose-6-phosphate (Δ*E_G*6*P_* = *E*2 − *E*1) or glucose (Δ*E_Glc_* = *E*3 − *E*2) and the dilution factor (*D*) were applied in formula 1 to calculate the concentration of D-glucose and *G*6*P*. Primary addition of the *G*6*P* dehydrogenase and the intermediate measurement (*E*2) depicted the formation of NADH by an enzymatic conversion of *G*6*P*, while adding the hexokinase glucose was initially converted by a two-step enzymatic reaction.

(1) $$G6P\left[\frac{g}{L}\right]=\left(\left(\frac{\left(V\times Mw\right)}{\left(\varepsilon_{340}\times d\times v\right)}\right)\times \Delta E\right)\times D$$

### 4.5. Membrane Isolation and Enzyme Kinetics

Cells were cultivated in serum bottles (1 L) to isolate membranes containing active NQR. Cells were harvested by centrifugation (9000× *g*, 30 min, 4 °C) and washed twice in 2 mM Tris-H_2_SO_4_, pH 7.5 with 50 mM K_2_SO_4_. 10 g of cells were suspended in 30 mL of 20 mM Tris-H_2_SO_4_ (pH 7.5) containing 50 mM K_2_SO_4_, 5 mM MgSO4, 1 mM dithiothreitol, 1 mM phenylmethylsulfonyl fluoride (PMSF), 0.1 mM di-isopropyl fluorophosphate and traces of DNase I (Roche, Basel, Switzerland). The suspension was passed three times through an EmulsiFlex^®^-C3 high-pressure homogenizer (Avestin, Mannheim, Germany) at 20,000 psi. Cell debris and unbroken cells were removed by centrifugation at 27,000× *g* for 30 min and at 4 °C. Membranes were collected by ultracentrifugation at 160,669× *g* for 90 min at 4 °C and washed once in 20 mM Tris-H_2_SO_4_ (pH 7.5) containing 50 mM K_2_SO_4_ and 5 % (*v*/*v*) glycerol and suspended in the same buffer [45].

Enzyme kinetic assays were carried out in a quartz cuvette (1 cm diameter) in a total volume of 1 mL at 25 °C using a Diode-Array spectrophotometer (Analytik Jena Specord S600, Jena, Germany). NADH oxidation was followed at 340 nm (*ε*_NADH_ = 6.22 mM^−1^ × cm^−1^), ubiquinone (Q1) reduction at 280 nm (*ε*_Q1_ = 14.5 mM^−1^ × cm^−1^) [46] and DMN reduction at 270–290 nm (*ε*_DMN_ = 15.2 mM^−1^ × cm^1^) [47]. The assays were conducted in buffer containing 20 mM Tris-H_2_SO_4_ (pH 7.5), 100 mM Na_2_SO_4_, 100 µM quinone, 100 µM NADH and ~100 µg protein.

### 4.6. Fluorescence Microscopy

Cells of *P. bryantii* B_1_4 were cultivated in Hungate tubes and incubated with 0 and 20 µM monensin up to 73 h. Cells were harvested by sampling 0.5 mL after 24, 48 and 73 h of incubation by centrifugation at 4 °C with 1000× g for 5 min. Cells were washed for 15 min in phosphate buffered saline (PBS) and fixed by shaking for 2 h at 300 rpm in 4% *p*-formaldehyde at 4 °C. Afterwards, cells were washed with ultrapure water and stained with 4 µg/mL SYTO^TM^ 9 and 100 µg/mL Alexa Fluor 594^TM^ Concanavalin A conjugate (ConA-594; both Invitrogen, Waltham, MA, USA) for 30 min at room temperature in the dark. Stained cells were gently washed with ultrapure water to remove unbound dye. Finally, cells were suspended in 50 µL ultrapure water, were mounted on glass slides without cover slip, and air-dried.

The biofilms were visualized by a confocal laser scanning microscope (Zeiss LSM 800) and using a non-immersion objective EC Epiplan-Apochromat 50x/0.95 HD DIC M27. The used laser wavelengths were 488 nm for SYTO^TM^ 9 and 561 nm for ConA-594. Images were recorded with a scan speed of two and an averaging number of two. The ZEN Blue 2.6 imaging software (Carl Zeiss GmbH, Jena, Germany) was used and images were exported as JPEG format.

### 4.7. Proteome Analysis Preparation

Steps of protein extraction, SDS-PAGE, in-gel digest and STAGE tips were performed as explained in Trautmann et al. [39]. Dried peptides were suspended in 0.1% formic acid before tandem mass spectrometry.

### 4.8. Tandem Mass Spectrometry

Peptide separation by liquid chromatography and mass spectrometry (MS) analysis were conducted via a Nano-LC-ESI-MS/MS using an Ultimate 3000 RSLC nano system (Dionex, Thermo Fisher Scientific, Darmstadt, Germany) coupled to a Q-Exactive HF-X mass spectrometer (Thermo Fisher Scientific, Darmstadt, Germany) using an EASY-Nano Flex ion source (Thermo Fisher Scientific, Darmstadt, Germany) in the Core Facility module mass spectrometry (University of Hohenheim, Stuttgart, Germany). Tryptic digested peptides were injected and passed through a precolumn (µ-precolumn C18 PepMap100, 300 µm, 100 Å, 5 µm × 5 mm, Thermo Fisher Scientific, Darmstadt, Germany) and a NanoEase analytical column (NanoEase M/Z HSS C18 T3, 1.8 µm 100 Å 75 µm × 250 mm column, Waters GmbH, Eschborn, Germany). All operations were run at a constant temperature of 35 °C and a flow of 300 nL/min. A gradient with solvent A (0.1% formic acid) and B (0.1% formic acid and 80 % acetonitrile) was run for 110 min (0–3–34–67–90–110 min) with increasing solvent B (0–2–15–30–45–95%) and a subsequent isocratic rinsing for 15 min and an equilibration from 95 to 2% within 10 min. Orbitrap detected at a resolution of 60.000 with 200 *m*/*z*, while the survey spectra were performed in a range from 200–2000 *m*/*z*. Tandem MS spectra were generated for the 30 most abundant peptide precursors using high energy collision dissociation (HCD) at a resolution of 15,000, normalized by a collision energy of 27. Lock-mass ions from the ambient air were used for internal calibration [48].

### 4.9. Proteome Analysis

The obtained tandem mass spectra were used for label-free quantification (LFQ) in MaxQuant (v.1.6.0.16) [49]. Two proteomic datasets were analyzed in separate: first *P. bryantii* B_1_4 exposed to a monensin concentration gradient after 9 h incubation time and second *P. bryantii* B_1_4 grown in absence or presence of monensin in a time series experiment. As a reference database *P. bryantii* B_1_4 proteins from Uniprot (3757 proteins entries, 12/2019) were used for both searches. Re-quantification was enabled, specific cleavage via trypsin and using only oxidation as a possible modification was set in the adjustments.

Protein quantification within a single cultivation condition was defined by the mean of at least four positive LFQ-values (>0) out of six replicates in the concentration gradient approach or at least two positive LFQ-values out of triplicates in the time series approach. Protein comparison was performed using the fold-change difference by setting the culture without monensin equal to one in the dataset of monensin gradient experiment or setting the incubation time of 9 h to one in time series experiment. If the fold-change of those references was zero, the lowest LFQ average of the different treatments was set equal to one. Two samples with low protein and peptide counts were excluded for fold-change analysis. Functional classification by Kyoto Encyclopedia Genes and Genome (KEGG) Orthologous groups (KO) of the identified proteins was conducted using EggNOG and Kofam, while last one was prioritized in case of discrepancies [50,51]. Proteins without any COG assignment were set manually as COG class S! (Function unknown). In total, 237 unknown proteins and 130 proteins from the COG class G, originating from protein identifications of proteomic dataset 2, were used in order to obtain specific information about carbohydrate-active enzymes (CAZymes) by using dbCAN2 with the HMMER tool [52]. Twenty-four uncharacterized proteins with a fold-change ≥5 were compared via NCBI’s protein-protein BLAST to the Uniprot/Swiss-Prot database to find homologous proteins with a described functional annotation. Models (XM/XP) and uncultured/environmental sample sequences were excluded in the blast search. Annotations with a maximal score ≥90 were considered as potential candidates for new characterization.

### 4.10. Statistical Analysis

Growth parameters (OD and pH), residual glucose concentration in the supernatant and intracellular Na^+^ concentration were examined regarding their significance between the used monensin concentrations and time points of measurement for the OD. Therefore, analysis of variance (ANOVA) and the least significant difference (LSD) test were performed by using Infostat (version 2018) [53]. Growth rate was determined by identifying the slope of a trend line, which was derived from five subsequent time points and their respective OD. Proteomics data were analyzed by a Bray Curtis similarity and principal component (PCO) analysis using the statistical software Primer 6 (v 6.1.16) and Permanova (v 1.0.6). Venn diagram for protein distribution among treatments was conducted using an interactive Venn diagram [54]. Analysis of fold-change and heat map illustrations were conducted using Excel 2016 (Microsoft Corporation, Redmond, Washington, DC, USA). The correlation coefficient (R²) was used to evaluate a connection among proteins or to other monitored parameters in Excel 2016.

## 5. Conclusions

In conclusion, a partially dose-dependent response of *P. bryantii* B_1_4 against monensin was identified with proteomics profiling, measurements of cellular compounds and enzyme activities as well as fluorescence microscopy of extracellular polysaccharides. The results showed a reduced growth and an enhanced Na^+^ efflux by elevated abundance of Na^+^-translocating proteins as a cellular response to the ionophore treatment. In addition, cells seem to evolve a protection mechanism by stripping their extracellular polysaccharide layer to get rid of the attached monensin. This can be hypothesized as a general resistance strategy against ionophore antibiotics. Further studies should confirm these findings and elucidate the concomitant impact of monensin-altered *Prevotella* sp. Towards other members of the rumen microbiome.

## Data Availability

Raw mass spectrometric data are stored in the ProteomeXchange Consortium including the data set identifier PXD026085 for the monensin concentration variants and PXD026057 for the time series [55]. Analyzed data are provided as supplementary information.

## Supplementary Materials

The following are available online at https://www.mdpi.com/article/10.3390/ijms221910202/s1.
