# Supplementary material for: Na+-Coupled Respiration and Reshaping of Extracellular Polysaccharide Layer Counteract Monensin-Induced Cation Permeability in Prevotella bryantii B14
Source: Int J Mol Sci. 2021 Sep 22;22(19):10202. doi: 10.3390/ijms221910202 (PMC8508442; doi:10.3390/ijms221910202)
Supplement: Supplementary file 1 [file ijms-22-10202-s001.zip › 20210827 Supplementary Material.pdf]

SUPPLEMENTARY MATERIAL

Na^+-coupled respiration and reshaping of extracellular polysaccharide layer counteract monensin-induced cation permeability in *Prevotella bryantii* B14

Trautmann, A.[1,2]; Schleicher, L.[1,3]; Pfirrmann, J.[2]; Boldt, C.[4]; Steuber, J.[1,3]; Seifert, J.[1,2,*]

[1] HoLMiR - Hohenheim Center for Livestock Microbiome Research, University of Hohenheim, Stuttgart, Germany

[2] Institute of Animal Science, University of Hohenheim, Stuttgart, Germany

[3] Institute of Biology, University of Hohenheim, 70599 Stuttgart, Germany

[4] Institute of Bioscience, TU Bergakademie Freiberg, 09599 Freiberg, Germany

* Corresponding author: jseifert@uni-hohenheim.de

Jana Seifert

University of Hohenheim

Institute of Animal Science

Emil-Wolff-Str. 6-10

70593 Stuttgart, Germany

TABLES:

Table S1. Protein and peptide count from replicates among monensin treatments.
A to F indicate the replicates while the treatments with monensin are given from 0 to 50 μM. Count of proteins represents the number of proteins that were quantified. Count of peptides indicate number of quantified peptides.

Monensin [μM]	A	B	C	D	E	F	
0	1,130	1,141	985	1,041	1,070	1,096	Proteins
10	1,321	1,323	1,349	1,303	1,294	1,286	
20	1,248	1,292	1,299	1,289	1,287	1,283	
50	1,289	1,274	1,307	1,290	1,294	1,290	
0	13,352	13,936	10,861	12,154	12,464	12,463	Peptides
10	14,969	14,620	14,792	14,818	14,764	14,560	
20	13,212	14,954	14,992	14,611	14,608	14,473	
50	14,573	14,044	14,862	14,608	14,586	14,328	

Table S2. Differences in clusters of orthologous group (COG) classes depending on 0, 10, 20 or 50 μM monensin. Mean of proteins (n=6) belonging to the displayed COG classes was standardized by total and compared for each concentration. COGs with a difference ≥1% were displayed in red lettering. Cultivation parameters were attached below and display the mean of six replicates. Intermediate columns display with a reddish color an elevation and with bluish color a decrease of functional class proteins under monensin supplementation. A least significant difference (LSD, p<0.01) test was performed for all displayed COG classes and cultivation parameters (small letters in right columns).

(COG) Description	Mean Contribution				Differences to 0 μM			Fisher LSD (p<0.01)			
	0 μM	10 μM	20 μM	50 μM	10 μM	20 μM	50 μM	0 μM	10 μM	20 μM	50 μM
(C) Energy production and conversion	7.6%	8.7%	9.2%	8.8%	1.14%	1.65%	1.28%	a	b	b	b
(D) Cell cycle control, cell division, chromosome partitioning	0.1%	0.4%	0.4%	0.4%	0.30%	0.36%	0.34%	a	b	c	c
(E) Amino acid transport and metabolism	4.7%	4.3%	4.6%	4.6%	-0.36%	-0.06%	-0.11%	a	b	ab	ab
(F) Nucleotide transport and metabolism	3.6%	3.9%	3.8%	3.9%	0.26%	0.21%	0.31%	a	b	ab	b
(G) Carbohydrate transport and metabolism	15.3%	14.4%	15.3%	15.5%	-0.86%	-0.02%	0.18%	-	-	-	-
(H) Coenzyme transport and metabolism	1.6%	1.1%	1.1%	1.2%	-0.46%	-0.44%	-0.42%	a	b	b	b
(I) Lipid transport and metabolism	1.8%	1.7%	1.7%	1.6%	-0.12%	-0.13%	-0.17%	-	-	-	-
(J) Translation, ribosomal structure and biogenesis	6.2%	24.4%	25.5%	26.3%	18.20%	19.27%	20.10%	a	b	b	b
(K) Transcription	0.6%	2.8%	3.2%	3.0%	2.16%	2.55%	2.39%	a	b	c	bc
(L) Replication, recombination and repair	0.3%	1.1%	1.2%	1.2%	0.83%	0.89%	0.90%	a	b	b	b
(M) Cell wall/membrane/envelope biogenesis	16.9%	8.7%	7.7%	7.5%	-8.19%	-9.17%	-9.36%	a	b	b	b
(N) Cell motility	0.8%	0.9%	1.0%	0.9%	0.08%	0.16%	0.11%	-	-	-	-
(O) Post-translational modification, protein turnover, and chaperones	7.1%	9.9%	8.5%	8.7%	2.82%	1.44%	1.59%	a	c	b	b
(P) Inorganic ion transport and metabolism	7.2%	1.9%	1.9%	1.9%	-5.31%	-5.35%	-5.36%	a	b	b	b
(Q) Secondary metabolites biosynthesis, transport, and catabolism	0.0%	0.0%	0.1%	0.1%	0.02%	0.03%	0.03%	a	b	b	b
(S) Function unknown	16.9%	9.0%	8.4%	8.0%	-7.88%	-8.50%	-8.82%	a	b	b	b
(!S) Function unknown (manually assigned)	8.7%	4.0%	3.6%	3.6%	-4.74%	-5.14%	-5.13%	a	b	b	b
(T) Signal transduction mechanisms	0.2%	0.6%	0.7%	0.7%	0.36%	0.44%	0.47%	a	b	c	c
(U) Intracellular trafficking, secretion, and vesicular transport	0.3%	1.8%	1.8%	1.7%	1.50%	1.53%	1.41%	a	b	b	b
(V) Defense mechanisms	0.1%	0.2%	0.3%	0.3%	0.17%	0.20%	0.19%	a	b	c	bc

Cultivation Parameters	Mean				Differences to 0 μM			Fisher LSD (p<0.01)			
	0 μM	10 μM	20 μM	50 μM	10 μM	20 μM	50 μM	0 μM	10 μM	20 μM	50 μM
**Optical density** [-] at 600 nm	1.83	1.30	0.98	0.89	-0.53	-0.85	-0.94	a	b	c	c
**pH** [$-\log_{10}[H^+]$] in supernatant	5.25	5.70	5.92	6.08	0.44	0.67	0.82	a	b	c	c
**Glucose** [g/L] in supernatant	0.06	0.24	0.90	0.77	0.18	0.84	0.71	a	a	b	b
**intracellular Na^+** per Protein [μg/mg]	128	33	59	105	-95	-69	-23	a	c	bc	ab

Table S3. Outer membrane (OM) related proteins under different monensin concentrations.
Protein abundances are illustrated as a heat map with a fold-change indicated by the color code (below heat map) and standardized by 0 µM monensin (set equal to one). Protein names are listed with the Uniprot ID in brackets.

Name (Uniprot ID)	10 µM	20 µM	50 µM
Starch-binding associating with OM (A0A1H9C300)	0.03	0.02	0.03
OM receptor for ferrienterochelin and colicins (D8E003)	0.06	0.08	0.06
Starch-binding associating with OM (D8DV05)	0.09	0.06	0.09
OM protein assembly factor BamA (D8DWS2)	0.14	0.07	0.10
OM receptor proteins, mostly Fe transport (A0A1H9CY22)	0.15	0.11	0.09
OM protein, cobalt-zinc-cadmium efflux system (A0A1H9FFT0)	0.14	0.11	0.11
OM protein (D8DXY2)	0.11	0.12	0.14
OM protein (D8DXY1)	0.11	0.12	0.14
OM protein beta-barrel domain-containing protein (D8DZT1)	0.21	0.13	0.12
TonB-linked OM protein, SusC/RagA family (A0A1H9B8Y5)	0.14	0.13	0.14
OM protein transport protein (Ompp1/fadl/todx) (D8DTK4)	0.18	0.15	0.13
TonB-dependent OM receptor (D8DUK7)	0.15	0.20	0.23
Cationic OM protein OmpH (D8DT44)	0.32	0.15	0.15
OM protein beta-barrel family protein (A0A1H8Z174)	0.17	0.16	0.16
OM autotransporter barrel domain-containing protein (D8DU39)	0.26	0.19	0.17
Starch-binding associating with OM (A0A1H9E645)	0.23	0.18	0.19
TonB-linked OM protein, SusC/RagA family (A0A1H9C2R2)	0.24	0.21	0.18
TonB-linked OM protein, SusC/RagA family (A0A1H9LDK0)	0.27	0.23	0.19
OM protein TolC (D8DWK0)	0.31	0.21	0.22
OM protein beta-barrel domain-containing protein (D8DYY2)	0.41	0.25	0.21
TonB-linked OM receptor P92 (D8DTK2)	0.37	0.26	0.22
Efflux transporter, OM factor (OMF) lipoprotein, NodT family (D8DVS0)	0.28	0.24	0.23
Starch-binding associating with OM (D8DUK8)	0.23	0.32	0.41
Major OM protein OmpA (D8DWD3)	0.27	0.24	0.25
OM protein (D8DZK0)	0.34	0.27	0.24
OM receptor proteins, mostly Fe transport (D8E0H5)	0.31	0.27	0.25
OM protein beta-barrel domain-containing protein (D8DWZ7)	0.26	0.25	0.27
OM receptor proteins, mostly Fe transport (A0A1H9DV15)	0.26	0.30	0.31
OM protein OmpA (A0A1H9DD30)	0.40	0.29	0.28
OM protein beta-barrel domain-containing protein (D8DVA3)	0.36	0.30	0.00
Cationic OM protein OmpH (D8DT45)	0.52	0.33	0.36
OM protein beta-barrel domain-containing protein (D8DWQ2)	0.43	0.33	0.46
OM efflux protein (D8DYZ4)	0.47	0.38	0.34
OM protein (D8DT53)	0.35	0.47	0.53
Periplasmic chaperone for OM proteins Skp (D8DT43)	0.55	0.39	0.39
OM protein beta-barrel domain-containing protein (D8DUD8)	0.60	0.48	0.50
OM lipoprotein-sorting protein (A0A1H9GE28)	0.75	0.64	0.59
OM lipoprotein carrier protein LolA (D8DTI7)	0.78	0.64	0.60
Periplasmic chaperone for OM proteins Skp (D8DSW6)	1.32	1.17	1.20

**Color Code:**	0.10	0.50	1.00

Table S4. Transport related proteins under different monensin concentrations.
Protein abundances are illustrated as a heat map with a fold-change indicated by the color code (below heat map). Protein names are listed with the Uniprot ID in brackets. Bluish marked proteins (upper part) indicate protein elevated in absence of monensin while reddish marked proteins (lower part) show increased abundance under monensin presence.

Name (Uniprot ID)	0 µM	10 µM	20 µM	50 µM
Putative auto-transporter adhesin, head GIN domain (D8DVY5)	1.00	0.15	0.08	0.06
Outer membrane receptor proteins, mostly Fe transport (A0A1H9CY22)	1.00	0.15	0.11	0.09
Outer membrane protein transport protein (D8DTK4)	1.00	0.18	0.15	0.13
Long-chain fatty acid transport protein (A0A1H9JWM8)	1.00	0.19	0.19	0.15
Outer membrane autotransporter protein (D8DU39)	1.00	0.26	0.19	0.17
Efflux transporter, outer membrane factor lipoprotein (D8DVS0)	1.00	0.28	0.24	0.23
Outer membrane receptor proteins, mostly Fe transport (D8E0H5)	1.00	0.31	0.27	0.25
Outer membrane receptor proteins, mostly Fe transport (A0A1H9DV15)	1.00	0.26	0.30	0.31
Long-chain fatty acid transport protein (D8DZW6)	1.00	0.44	0.34	0.31
Outer membrane receptor proteins, mostly Fe transport (D8DSX8)	1.00	0.00	0.00	0.00
Outer membrane receptor proteins, mostly Fe transport (D8DTP4)	1.00	0.00	0.00	0.00
Outer membrane receptor proteins, mostly Fe transport (D8DZ53)	1.00	0.00	0.00	0.00
Ferrous iron transport protein B (D8DT71)	1.00	15.92	17.06	15.32
Phospholipid/cholesterol/gamma-HCH transport system (A0A1H8YR75)	1.00	4.87	4.69	4.62
ABC transporter, ATP-binding protein (D8DYR5)	1.00	3.04	3.25	3.14
ABC transporter, ATP-binding protein (D8DZW0)	1.00	2.81	3.30	3.22
Sodium/glucose cotransporter (D8DYQ7)	1.00	2.22	3.00	3.12
ABC-type amino acid transport/signal transduction system (D8DYU7)	1.00	2.08	2.89	2.71
Biopolymer transport protein ExbB (D8DVL9)	1.00	2.09	2.60	2.63
Hexuronate transporter (D8DVQ2)	1.00	2.11	2.61	2.62
Electron transport complex protein RnfG (D8DXV4)	1.00	2.08	2.41	2.73
Electron transport complex protein RnfG (D8DYE1)	1.00	2.82	1.93	2.37
Di-tripeptide ABC transporter (D8DWZ5)	1.00	2.00	2.13	2.07
Biopolymer transport protein ExbD/TolR (D8E014)	1.00	1.94	1.84	1.67
Glutamine ABC transporter, ATP-binding protein GlnQ (D8DYU8)	1.00	1.92	1.92	1.84
Cell division transport system ATP-binding protein (D8DY89)	1.00	1.65	2.21	1.90
NitT/TauT family transport system substrate-binding protein (D8DY75)	1.00	1.46	1.77	1.65
Phosphate transport system substrate-binding protein (D8E016)	1.00	1.36	1.39	1.40
Biopolymer transport protein, ExbD/TolR family (D8E013)	1.00	1.12	1.05	1.08
Maltose/moltooligosaccharide transporter (D8DXY5)	0.00	1.00	1.65	1.82
Transporter (D8DT67)	0.00	1.00	1.53	1.89
MFS transporter, FHS family, L-fucose permease (D8DYR0)	0.00	1.21	1.26	1.00
ABC-type sugar transport system (D8DUK6)	0.00	1.00	1.19	1.25
Ca2+-transporting ATPase (D8DXJ6)	0.00	1.00	1.30	1.10
ABC transporter, ATP-binding protein (D8E0H6)	0.00	1.00	1.35	1.05
Putative ABC transport system permease protein (A0A1H9F867)	0.00	1.00	1.32	1.03
Putative transport protein (A0A1H9JA50)	0.00	1.00	1.23	1.08
ABC transporter, ATP-binding protein, MsbA family (D8DZU9)	0.00	1.00	1.23	1.04
Na+/H+ antiporter NhaD (D8DYG5)	0.00	1.00	1.16	1.11
Phosphate:Na+ symporter (A0A1H9BJD7)	0.00	1.05	1.20	1.00
ABC transporter, ATP-binding protein (D8DSX4)	0.00	1.00	1.16	1.07
ABC transporter domain protein (D8DWC8)	0.00	1.00	1.11	1.08
ABC transporter, ATP-binding protein (D8DWW0)	0.00	1.04	1.00	1.14
ABC transporter domain protein (D8DY72)	0.00	1.00	1.12	1.03
ABC transporter, ATP-binding protein (D8DZ98)	0.00	1.00	1.08	1.03
Kef-type K+ transport system, membrane component KefB (D8DZH8)	0.00	0.00	1.09	1.00
Zinc ABC transporter, ATP-binding protein ZnuC (D8DT91)	0.00	0.00	1.05	1.00
Cation ABC transporter, periplasmic-binding protein (D8DT92)	0.00	0.00	0.00	1.00

Color Code:	0.00	0.10	2.00	5.00

Table S5. Proteins with highly correlating intracellular sodium. Proteins abundances (natural logarithm of LFQ values) are displayed in color code to show also the magnitude of differences between the proteins. Only proteins with correlations ≥0.7 are shown. Intracellular Na^+ concentration is presented in last row.

**Name (Uniprot ID)**	**Monensin**				**Correlation**
	**0 µM**	**10 µM**	**20 µM**	**50 µM**	**with Na^+**
N-carbamoylputrescine amidase (A0A1H9EHL1)	20.32	20.83	20.74	20.68	-0.74
ATP-dependent Clp protease ATP-binding subunit ClpX (A0A1H8YR68)	18.46	21.18	21.19	21.03	-0.70
Beta-glucosidase (D8DY03)	20.70	19.08	19.37	19.61	0.70
TonB-dependent receptor, plug (D8DVB1)	20.73	19.13	19.52	19.77	0.71
Putative lipoprotein (D8DU09)	21.17	19.70	20.19	20.35	0.72
IPT/TIG domain-containing protein (A0A1H9LFB0)	22.19	20.99	21.36	21.42	0.73
RagB/SusD domain protein (D8DT52)	22.05	20.86	21.31	21.44	0.74
IPT/TIG domain-containing protein (D8DY17)	23.19	22.10	22.29	22.36	0.76
TonB-dependent receptor, plug (D8DY14)	20.92	19.88	20.17	20.28	0.78
Putative lipoprotein (D8DY15)	22.42	21.49	21.85	21.91	0.78
Outer membrane protein (D8DT53)	19.95	19.51	19.50	19.70	0.79
Extracellular Na^+ standardized by protein [µg/mg]	128	33	59	105	1.00

Color Code: 18 | 19 | 21 | 23

Table S6. NADH:Quinone Reductase (NQR) in a time series with and without monensin.

Name (Uniprot ID)	0 µM Monensin (Con)				20 µM Monensin (Mon)				Ratio Mon : Con			
	9h	24h	48h	72h	9h	24h	48h	72h	9h	24h	48h	72h
NQR subunit A (D8DWC1)	1.00	7.76	11.49	11.83	1.00	1.54	1.83	2.54	3.68	0.73	0.59	0.79
NQR subunit B (D8DWC0)	-	1.00	1.65	1.89	1.00	1.13	1.63	2.79	Mon	0.99	0.86	1.29
NQR subunit B (D8DWP0)	1.00	2.36	4.69	4.41	1.00	1.11	1.49	1.92	4.27	2.01	1.35	1.86
NQR subunit C (D8DWB9)	1.00	6.14	8.87	10.10	1.00	1.28	1.56	2.07	3.57	0.74	0.63	0.73
NQR subunit F (D8DWB6)	1.00	6.86	9.88	8.73	1.00	1.37	1.57	1.50	13.45	2.69	2.13	2.30
NQR subunit H (D8DWN8)	-	1.00	-	-	1.00	1.56	2.30	-	Mon	0.98	Mon	-
NQR subunit I (D8DWN7)	1.00	5.53	9.30	10.04	1.00	1.17	1.29	2.09	6.34	1.34	0.88	1.32
NQR subunit J (D8DWN6)	-	1.00	-	-	1.00	1.42	2.05	-	Mon	0.73	Mon	-
NQR subunit L (A0A1H9A8K0)	-	1.00	-	-	1.00	1.81	3.38	4.04	Mon	1.24	Mon	Mon
NQR subunit M (A0A1H9A8A6)	-	-	-	-	-	1.00	2.02	-	-	Mon	Mon	-
**Color code:**	0	1	2	5	10	**Color code:**	Con	0.5	1	5	Mon	

Table S7. pBLAST results of uncharacterized proteins ranked by best score combined with fold change.
Left part illustrates the fold change of identified uncharacterized proteins. Color code is below the table. Right part shows the candidate matches with the best score and other parameters.

Name (Uniprot ID)	Monensin				pBLAST							
	0 µM	10 µM	20 µM	50 µM	Lenght	Max Score	E-value	Query Cover	% Ident	Protein Name	Acession No.	Organism
UP, UPF0371 family (D8DTQ4)	1.00	16.25	19.68	17.55	595	576	0.0E+00	0.98	56.9	UPF0371 protein cu0538 [Corynebacterium...	B1VFF9.1	*Corynebacterium*
UP (D8DVZ9)	1.00	10.53	13.16	13.49	210	140	3.0E-41	0.96	31.6	Protein YghO [Escherichia coli K-12]	Q46840.3	*Escherichia coli* K-12
UP (D8DYX8)	1.00	0.33	0.17	0.15	197	130	1.0E-35	0.75	33.3	Radial spoke head 10 homolog B [Danio rerio]	Q08CH7.1	*Danio rerio* (Zebra fish)
UP (D8DZR0)	1.00	0.23	0.18	0.19	318	121	4.0E-33	0.88	19.6	Uncharacterized protein aq_1259; Flags: Precurso...	O67300.1	*Aquifex aeolicus* VF5
UP (A0A1H8YRX1)	1.00	4.47	5.17	5.26	490	96	7.0E-26	0.84	23.0	Uncharacterized protein YitL [Bacillus subtilis...	O06747.2	*Bacillus subtilis* subsp. *subtilis* str. 168
UP: Uncharacterized protein	0.00	0.10	0.25	5.00	15.00	**Color Code**						

Table S8. Protein and peptide count from replicates among 0 and 20 μM monensin cultivations over time. A, B, C indicate the replicates while time points reach from 9, 24, 48 to 72 h. Count of proteins represents the number of proteins that were quantified. Count of peptides indicate number of quantified peptides. Red numbers indicate low counts and were excluded for fold-change calculations.

Time [h]	Con (0 μM)			Mon (20 μM)			
	A	B	C	A	B	C	
9	903	941	896	1,339	1,383	1,380	Proteins
24	1,089	1,117	1,016	1,258	1,290	1,293	
48	646	1,006	905	1,300	1,280	1,285	
72	891	1,008	846	1,146	1,027	532	
9	8,721	10,350	9,508	14,575	14,609	14,331	Peptides
24	10,869	12,210	10,191	13,831	14,011	14,070	
48	4,184	9,581	7,121	13,021	12,725	12,963	
72	6,917	10,139	6,537	9,389	7,484	3,811	

Table S9. Differences in COG classes and cultivation parameters at 9, 24, 48 and 72 h of cultivation with 20 µM monensin or without.
Mean occurrence of proteins (n=3 and for control 48 h and monensin 72 h) belonging to the displayed COG classes were standardized by total and compared for each time point. COG classes with red font were mostly affected (>1 %) at a time point in their abundance between Con and Mon, displayed in the right column with blue (more abundant in Mon) and red (more abundant in Con). Cultivation parameters were attached below and display the mean of triplicates. For all displayed parameters significance with respect to cultivation condition over all time points (* in front of parameter description) and time (displayed by different small letters in right columns) was determined with p<0.05.

(COG) Description	Control [0 µM]				Monensin [20 µM]				Differences (Con-Mon)				Time (p<0.05)			
	9h	24h	48h	72h	9h	24h	48h	72h	9h	24h	48h	72h	9h	24h	48h	72h
*** (C) Energy production and conversion**	3.5%	7.9%	7.8%	8.4%	8.2%	9.6%	9.8%	9.1%	-4.7%	-1.7%	-2.0%	-0.7%	a	b	b	b
* (D) Cell cycle control, cell division, chromosome partitioning	0.0%	0.1%	0.1%	0.1%	0.3%	0.4%	0.4%	0.3%	-0.3%	-0.3%	-0.3%	-0.2%	a	b	b	a
*** (E) Amino acid transport and metabolism**	2.6%	3.5%	3.6%	3.9%	4.2%	4.2%	4.3%	3.1%	-1.6%	-0.7%	-0.7%	0.8%	a	b	b	b
*** (F) Nucleotide transport and metabolism**	1.2%	2.6%	2.6%	2.8%	3.1%	3.7%	3.8%	3.1%	-1.9%	-1.1%	-1.2%	-0.3%	a	b	b	b
*** (G) Carbohydrate transport and metabolism**	12.0%	15.6%	12.5%	12.1%	16.0%	17.0%	16.5%	14.8%	-4.0%	-1.4%	-4.0%	-2.6%	a	b	ab	a
* (H) Coenzyme transport and metabolism	0.5%	1.0%	0.9%	0.9%	1.0%	1.1%	1.1%	0.7%	-0.5%	-0.1%	-0.3%	0.2%	a	b	b	b
(I) Lipid transport and metabolism	1.8%	2.0%	1.7%	1.7%	1.9%	1.8%	1.8%	1.3%	-0.1%	0.2%	-0.1%	0.4%	a	a	ab	b
*** (J) Translation, ribosomal structure and biogenesis**	3.6%	15.0%	18.7%	19.1%	18.0%	22.2%	22.8%	24.4%	-14.4%	-7.2%	-4.1%	-5.3%	a	b	b	b
*** (K) Transcription**	0.2%	1.1%	1.2%	1.4%	1.9%	2.4%	2.7%	2.3%	-1.7%	-1.3%	-1.5%	-0.9%	a	b	b	b
* (L) Replication, recombination and repair	0.3%	0.5%	1.1%	1.3%	0.9%	1.2%	1.4%	1.4%	-0.5%	-0.7%	-0.3%	-0.1%	a	b	c	d
*** (M) Cell wall/membrane/envelope biogenesis**	16.6%	9.7%	9.4%	8.3%	9.6%	7.5%	6.5%	6.8%	6.9%	2.2%	2.9%	1.5%	a	b	b	b
* (N) Cell motility	1.3%	0.6%	1.7%	1.6%	0.9%	0.6%	0.9%	1.6%	0.3%	0.0%	0.8%	0.0%	a	b	a	a
*** (O) Post-translational modification, protein turnover, and chaperones**	8.3%	11.9%	14.3%	16.2%	10.1%	10.6%	10.5%	13.0%	-1.8%	1.3%	3.8%	3.2%	a	b	b	c
*** (P) Inorganic ion transport and metabolism**	13.0%	6.9%	5.5%	4.8%	3.9%	1.9%	1.7%	1.7%	9.2%	5.0%	3.7%	3.1%	a	b	b	b
(Q) Secondary metabolites biosynthesis, transport, and catabolism	0.2%	0.0%	0.0%	0.0%	0.1%	0.1%	0.1%	0.0%	0.1%	0.0%	-0.1%	0.0%	a	b	b	b
*** (S) Function unknown**	19.8%	11.7%	9.7%	9.2%	11.1%	8.1%	7.8%	5.8%	8.6%	3.5%	2.0%	3.4%	a	b	b	b
*** (S!) Function unknown [manually annotated]**	14.8%	8.3%	7.2%	6.4%	6.4%	3.9%	3.7%	3.6%	8.4%	4.4%	3.4%	2.8%	a	b	b	b
* (T) Signal transduction mechanisms	0.1%	0.2%	0.2%	0.2%	0.4%	0.6%	0.7%	0.4%	-0.3%	-0.4%	-0.4%	-0.2%	a	b	c	b
*** (U) Intracellular trafficking, secretion, and vesicular transport**	0.1%	1.1%	1.3%	1.4%	1.6%	2.5%	3.0%	2.7%	-1.5%	-1.4%	-1.7%	-1.3%	a	b	c	ab
* (V) Defense mechanisms	0.0%	0.1%	0.2%	0.2%	0.2%	0.3%	0.3%	0.2%	-0.2%	-0.1%	-0.1%	0.0%	a	b	b	c

Cultivaton Parameters [Unit]	Control [0 µM]				Monensin [20 µM]				Differences (Con-Mon)				Time (p<0.01)			
	9h	24h	48h	72h	9h	24h	48h	72h	9h	24h	48h	72h	9h	24h	48h	72h
* **Optical Density** [-] at 600 nm	1.95	2.09	2.11	2.12	1.67	1.60	1.41	1.35	0.28	0.50	0.70	0.77	-	-	-	-
* **pH** $[-\log_{10}[H^+]]$ of supernatant	5.01	4.96	4.96	4.97	5.35	5.15	5.18	5.14	-0.34	-0.19	-0.22	-0.17	a	b	b	b
* **Glucose** [g/L] in supernatant	0.00	0.00	0.01	0.01	0.26	0.25	0.61	0.78	-0.26	-0.25	-0.60	-0.77	a	a	ab	b
* **Glucose-6-phosphate** [mg/L] in supernatant	0.00	0.00	0.28	0.96	2.06	2.75	8.40	11.84	-2.06	-2.75	-8.12	-10.87	a	ab	ab	b

Table S10. Enzymes of the starch metabolism (ko00500) with gradually ascending or descending abundance.
The green-yellow-red color code illustrates the fold-change within conditions control (Con) and monensin (Mon), while blue-yellow-brown shows the ratio of protein abundance of Mon:Con. Fields without quantification are displayed by a grey-filled field with a dash. Fields with Mon or Con indicate that in this sample the protein was only quantified in the monensin (Mon) or the control (Con) condition. Protein names written in red point out enzymes with glucose as a product.

Protein Name (Uniprot ID)	0 µM Monensin (Con)				20 µM Monensin (Mon)				Ratio Mon : Con			
	9h	24h	48h	72h	9h	24h	48h	72h	9h	24h	48h	72h
Alpha-L-arabinofuranosidase (D8E0G9)	1.00	0.03	-	-	1.00	0.28	0.10	-	0.29	2.45	Mon	-
Alpha-glucosidase (D8DXY7)	1.00	0.08	0.00	-	1.00	0.42	0.27	0.12	0.35	1.87	176.36	Mon
Beta-galactosidase (D8DV08)	1.00	0.19	0.08	-	1.00	0.29	0.31	0.40	0.34	0.52	1.28	Mon
Putative glycosyl hydrolase, family 13 (D8DXY9)	1.00	0.11	-	0.00	1.00	0.36	0.13	0.14	0.24	0.75	Mon	12.96
Beta-glucosidase (A0A1H9IED8)	1.00	0.10	-	0.07	1.00	0.96	1.70	4.95	0.13	1.25	Mon	9.27
Fructan beta-(2,6)-fructosidase (D8DUL1)	1.00	0.25	0.03	0.01	1.00	0.49	0.37	0.31	0.26	0.50	3.12	6.10
4-deoxy-L-threo-5-hexosulose-uronate ketol-isomerase (D8DVQ3)	1.00	0.14	0.05	0.05	1.00	0.29	0.31	0.36	0.48	0.99	2.89	3.22
Aldose 1-epimerase (A0A1H9EVQ9)	1.00	1.15	0.50	0.34	1.00	1.66	1.53	1.72	0.36	0.52	1.11	1.79
Beta-galactosidase (D8E0G2)	1.00	0.24	0.07	0.07	1.00	0.48	0.48	0.74	0.18	0.35	1.23	1.83
Transketolase (D8DYQ1)	1.00	6.12	6.50	6.76	1.00	1.62	1.78	1.49	3.19	0.85	0.87	0.71
Starch phosphorylase (A0A1H9J3G8)	1.00	8.48	12.87	13.26	1.00	1.60	1.71	1.84	5.25	0.99	0.70	0.73
4-alpha-glucanotransferase (D8DXY6)	1.00	16.19	24.25	25.60	1.00	2.37	3.01	2.33	6.62	0.97	0.82	0.60
Galacitol PTS, EIIc (D8DVQ4)	-	1.00	1.85	2.17	1.00	1.21	1.60	1.64	Mon	0.86	0.62	0.54
Hexuronate transporter (D8DVQ2)	-	1.00	1.29	1.47	1.00	1.72	1.59	1.09	Mon	0.97	0.69	0.42

Color code:	-	0.1	1	5	10

Color code:	Con	0.5	1	5	Mon

Table S11. Ratio of CAZyme families distribution between control and monensin out of 72 identified CAZymes. Depending if proteins of corresponding class were more abundant in control, monensin or in between, a related distribution was assigned depending on the intensity with an increasing number of plus signs (+). In families without a sufficient amount of total hits (<5), distribution assignation was omitted.

Enzyme Classes	Total Hits	Most found in condition			Most found in condition			Distribution
		Control	In between	Monensin	Control	In between	Monensin	
GH2	10	2	4	4	20%	40%	40%	+ Mon
GH43	6	4	1	1	67%	17%	17%	++ Con
GH13	5	0	1	4	0%	20%	80%	+++ Mon
PL1	5	4	0	1	80%	0%	20%	+++ Con
GH3	4	1	2	1	25%	50%	25%	-
GH51	4	0	2	2	0%	50%	50%	-
GH5	3	1	1	1	33%	33%	33%	-
GH97	3	1	0	2	33%	0%	67%	-
CE8	2	1	1	0	50%	50%	0%	-
GH106	2	0	0	2	0%	0%	100%	-
GH26	2	1	1	0	50%	50%	0%	-
GH28	2	2	0	0	100%	0%	0%	-
GH31	2	1	0	1	50%	0%	50%	-
GH32	2	1	1	0	50%	50%	0%	-
GH35	2	0	1	1	0%	50%	50%	-
GH53	2	1	1	0	50%	50%	0%	-
CBM20	1	0	1	0	0%	100%	0%	-
GH105	1	0	1	0	0%	100%	0%	-
GH127	1	0	0	1	0%	0%	100%	-
GH133	1	0	0	1	0%	0%	100%	-
GH141	1	0	1	0	0%	100%	0%	-
GH154	1	0	0	1	0%	0%	100%	-
GH163	1	0	1	0	0%	100%	0%	-
GH36	1	0	0	1	0%	0%	100%	-
GH57	1	0	0	1	0%	0%	100%	-
GH67	1	1	0	0	100%	0%	0%	-
GH78	1	0	0	1	0%	0%	100%	-
GH9	1	0	1	0	0%	100%	0%	-
GH94	1	0	0	1	0%	0%	100%	-
GT35	1	0	1	0	0%	100%	0%	-
GT5	1	0	0	1	0%	0%	100%	-
PL29	1	0	1	0	0%	100%	0%	-

Table S12. Cell division proteins (Fts) with and without monensin over time.
The green-yellow-red color code illustrates the fold-change within conditions control (Con) and monensin (Mon), while blue-yellow-brown shows the ratio of protein abundance of Mon:Con. Fields without quantification are displayed by a grey-filled field with a dash. Fields with Mon or Con indicate that in this sample the protein was only quantified in the monensin (Mon) or the control (Con) condition.

Name (Uniprot ID)	0 µM Monensin (Con)				20 µM Monensin (Mon)				Ratio Mon : Con			
	9h	24h	48h	72h	9h	24h	48h	72h	9h	24h	48h	72h
ATP-dependent zinc metalloprotease FtsH (D8DT63)	1.00	7.73	11.68	15.74	1.00	0.98	0.78	1.11	16.76	2.13	1.13	1.18
Cell division protein FtsA (D8DUA2)	-	1.47	1.42	1.00	1.00	1.56	1.74	1.04	Mon	4.12	4.75	4.04
Signal recognition particle receptor FtsY (D8DXW7)	-	1.22	1.00	1.04	1.00	1.27	1.63	0.95	Mon	2.91	4.58	2.55
Cell division protein FtsZ (D8DUA3)	-	1.00	1.65	1.50	1.00	1.11	1.32	1.44	Mon	3.06	2.20	2.64
Ftsk/spoiiie family protein (D8DYX1)	-	-	-	-	1.00	0.97	0.81	0.98	Mon	Mon	Mon	Mon
Cell division protein FtsI (Penicillin-binding protein 3) (D8DU94)	1.00	-	-	-	1.00	0.47	0.34	-	1.39	Mon	Mon	-
Cell division protein FtsX (D8DVR7)	-	-	-	-	-	1.00	1.42	3.81	-	Mon	Mon	Mon
Cell division protein FtsZ (D8DTR9)	-	-	-	-	-	-	1.00	-	-	-	Mon	-

Color code: - | 0.1 | 1 | 5 | 10   **Color code:** Con | 0.5 | 1 | 5 | Mon

Table S13. Glucose forming glucosidases with gradually descending abundance cells with and without monensin over time.
The green-yellow-red color code illustrates the fold-change within conditions control (Con) and monensin (Mon), while blue-yellow-brown shows the ratio of protein abundance of Mon:Con. Fields without quantification are displayed by a grey-filled field with a dash. Fields with Mon or Con indicate that in this sample the protein was only quantified in the monensin (Mon) or the control (Con) condition.

Name (Uniprot ID)	KO	0 µM Monensin (Con)				20 µM Monensin (Mon)				Ratio Mon : Con			
	Number	9h	24h	48h	72h	9h	24h	48h	72h	9h	24h	48h	72h
Alpha-glucosidase (D8DXY7)	K21574	1.00	0.08	0.00	-	1.00	0.42	0.27	0.12	0.35	1.87	176.36	Mon
Putative glycosyl hydrolase, family 13 (D8DXY9)	K21575	1.00	0.11	-	0.00	1.00	0.36	0.13	0.14	0.24	0.75	Mon	12.96
Beta-glucosidase (A0A1H9IED8)	K05349	1.00	0.10	-	0.07	1.00	0.96	1.70	4.95	0.13	1.25	Mon	9.27
Alpha-L-arabinofuranosidase A (D8DZD6)	K01209	1.00	0.11	0.08	0.05	1.00	0.33	0.30	0.19	0.59	1.87	2.12	2.36
Fructan beta-(2,6)-fructosidase (D8DUL1)	K03332	1.00	0.25	0.03	0.01	1.00	0.49	0.37	0.31	0.26	0.50	3.12	6.10
Beta-galactosidase (D8DV08)	K12308	1.00	0.19	0.08	-	1.00	0.29	0.31	0.40	0.34	0.52	1.28	Mon
Beta-galactosidase (D8E0G2)	K01190	1.00	0.24	0.07	0.07	1.00	0.48	0.48	0.74	0.18	0.35	1.23	1.83
Glycosyl hydrolases family 2 (D8DWD5)	K01190	1.00	0.44	0.40	0.45	1.00	0.56	0.54	0.74	0.43	0.55	0.57	0.71
Beta-galactosidase (D8DW02)	K01190	1.00	0.33	0.22	0.28	1.00	0.60	0.55	0.78	0.23	0.42	0.59	0.66

Color code: - | 0.1 | 1 | 5 | 10   **Color code:** Con | 0.5 | 1 | 5 | Mon

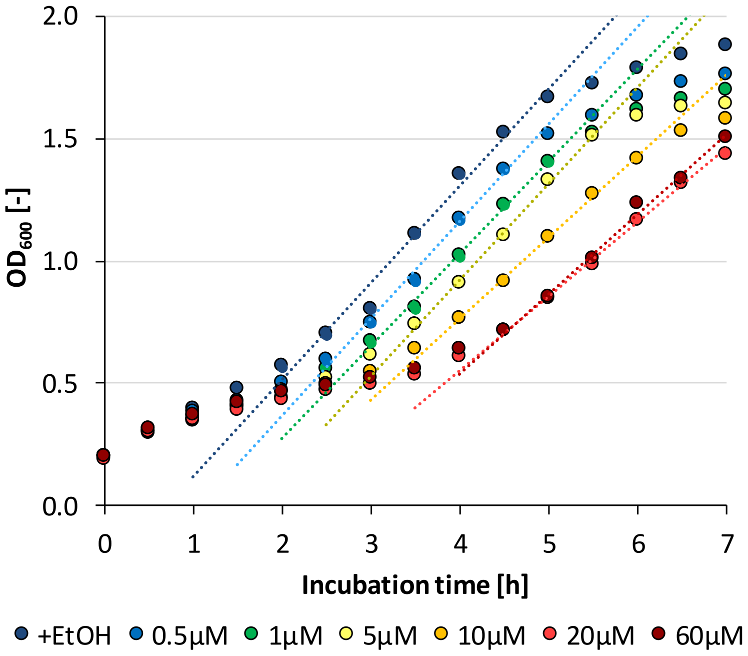

Condition	OD x h^{-1}
-EtOH	0.485
+EtOH	0.439
0.1 µM	0.401
0.5 µM	0.398
1 µM	0.378
2 µM	0.383
5 µM	0.393
10 µM	0.332
15 µM	0.336
20 µM	0.304
25 µM	0.313
30 µM	0.344
35 µM	0.338
40 µM	0.332
60 µM	0.326

Figure S1. Growth curves with table of growth rates per hour at various monensin concentrations. Average optical density (OD; n=3) was determined at a wavelength of 600 nm and a linear trend line was fitted through the measure points during the exponential growth phase. Table on the right side displays the growth rates during exponential phase of *P. bryantii* B14 under various monensin concentrations.

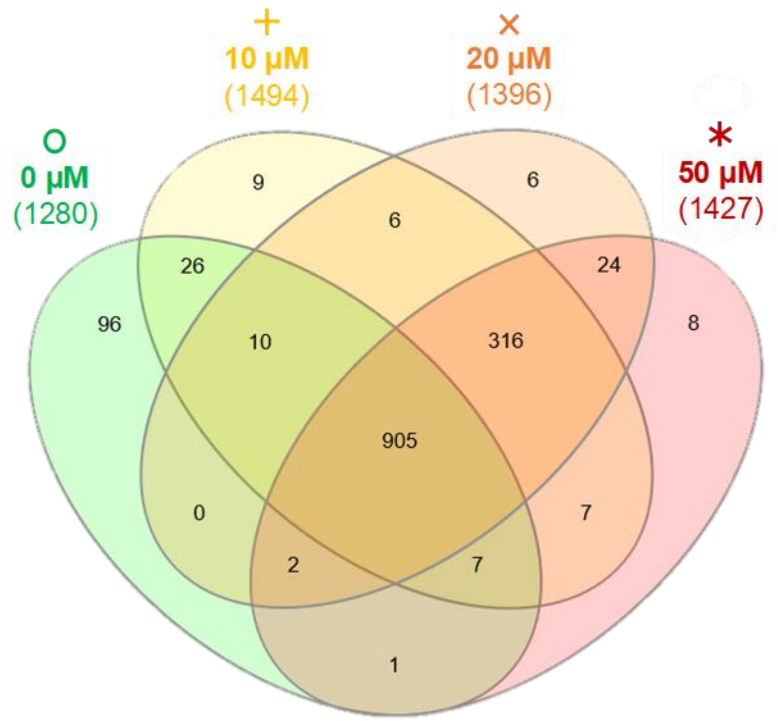

Figure S2. Venn diagram of proteomes under monensin. Each treatment contained six replicates (n=6). A protein was only counted if at least 4 of 6 replicates were quantified.

Time points				Control		Monensin	
9 h	24 h	48 h	72 h	n	%	n	%
O				136	11%	116	9%
	O			78	6%	18	1%
		O		5	0%	35	3%
			O	14	1%	2	0%
O	O			96	8%	44	4%
O		O		0	0%	17	1%
O			O	2	0%	5	0%
	O		O	30	2%	0	0%
		O	O	9	1%	3	0%
	O	O		14	1%	39	3%
O	O	O		21	2%	222	18%
O	O		O	28	2%	3	0%
O		O	O	1	0%	5	0%
	O	O	O	178	14%	15	1%
O	O	O	O	625	51%	942	76%
			**Total:**	**1237**		**1466**	

Figure S3. Dot plot of proteomes at 9, 24, 48 and 72 h of incubation time under control, monensin or combined conditions. Each time point at each condition consists of triplicates (n=3). Percental contribution to total number of quantified proteins for each condition and combination were given below the diagram.

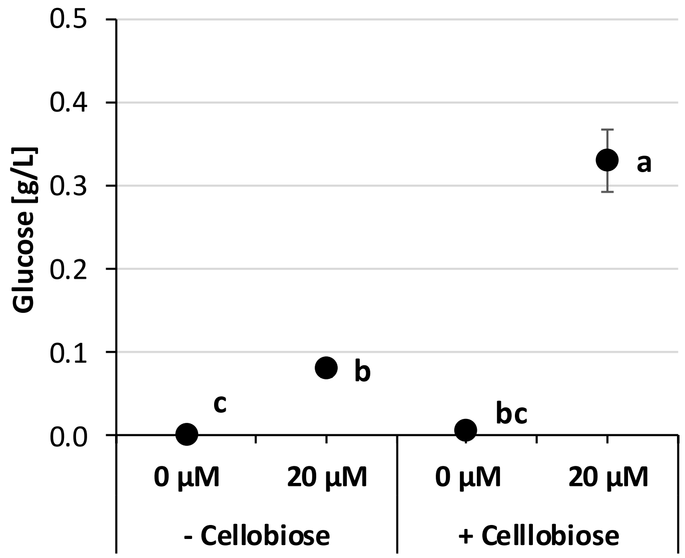

Figure S4. Glucose concentrations in supernatant of cultures with and without cellobiose and with and without monensin after 73 h. Black dots represent glucose with the left y-axis. Error bars indicate standard deviation. Not commonly shared letters indicate significant difference by the Fisher-LSD test ($p<0.01$) for glucose.

a

Name (Uniprot ID)	EC Number	0 µM Monensin (Con) 9h	24h	48h	72h	20 µM Monensin (Mon) 9h	24h	48h	72h	Ratio Mon : Con 9h	24h	48h	72h
ATP phosphoribosyltransferase (D8DYW0)	2.4.2.17	1.00	16.42	26.57	24.58	1.00	1.98	2.38	1.57	11.91	1.43	1.07	0.76
Histidine biosynthesis bifunctional protein HisIE (D8DY88)	3.5.4.19 / 3.6.1.31	-	2.18	1.44	1.00	1.00	1.28	1.47	0.82	Mon	0.65	1.13	0.91
1-phosphoribosylformimino-5-aminoimidazole-4-carboxamide isomerase (D8DY86)	5.3.1.16	1.00	0.10	-	0.14	1.00	0.45	0.47	-	0.18	0.78	Mon	Con
Imidazole glycerol phosphate synthase subunit HisH (D8DY85)	4.3.2.10 / 3.5.1.2	1.00	-	-	-	1.00	0.52	0.53	-	1.02	Mon	Mon	-
Imidazole glycerol phosphate synthase subunit HisF (D8DY87)	4.3.2.10	1.00	-	-	-	1.00	0.57	0.49	-	1.26	Mon	Mon	-
Histidine biosynthesis bifunctional protein HisB (D8DWS5)	3.1.3.15 / 4.2.1.19	-	-	-	-	1.00	1.74	2.59	1.91	Mon	Mon	Mon	Mon
Histidinol-phosphate aminotransferase (D8DYV8)	2.6.1.9	-	1.00	-	2.24	1.00	1.49	1.80	0.60	Mon	16.60	Mon	2.98
Histidinol dehydrogenase (D8DYV9)	1.1.1.23	-	1.00	-	-	1.00	0.99	1.24	0.87	Mon	8.04	Mon	Mon

Color code: - | 0.1 | 1 | 5 | 10

Color code: Con | 0.5 | 1 | 5 | Mon

b

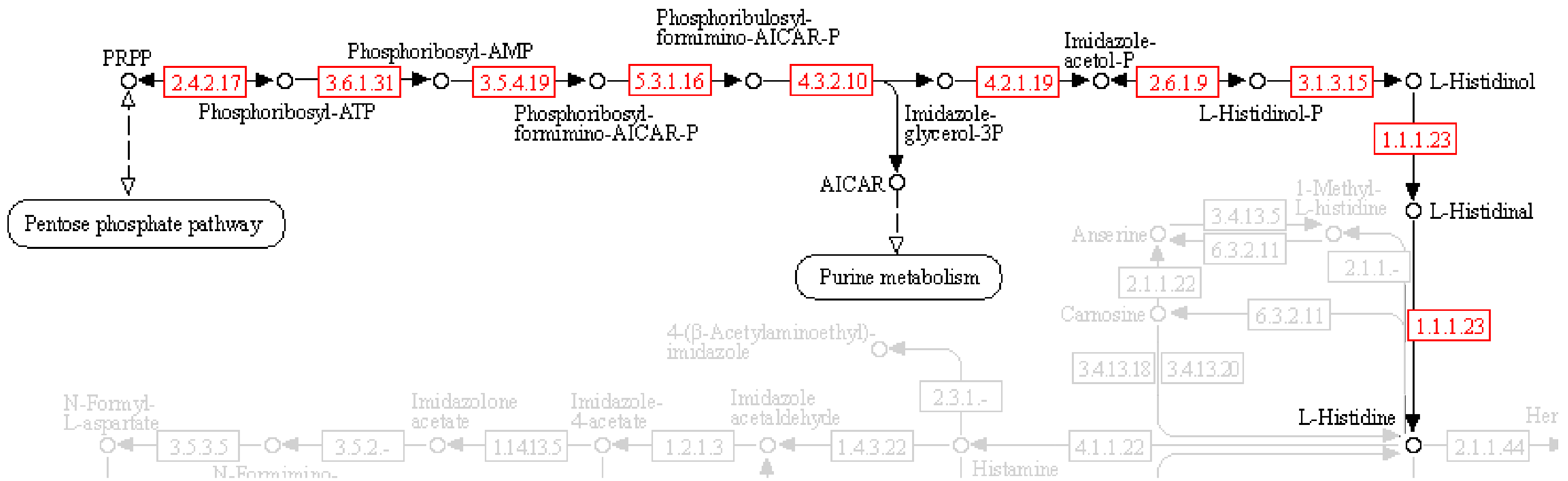

Figure S5. Histidine biosynthesis pathway with enzymes and their abundance within a time series between monensin absence or presence.
(**a**) Displays the abundance on respective enzymes in their order for histidine synthesis. The green-yellow-red color code illustrates the fold-change within conditions control (Con) and monensin (Mon), while blue-yellow-brown shows the ratio of protein abundance of Mon:Con. Fields without quantification are displayed by a grey-filled field with a dash. Fields with Mon or Con indicate that in this sample the protein was only quantified in the monensin (Mon) or the control (Con) condition. (**b**) The KEGG pathway (ko00350) for histidine biosynthesis. Red written EC numbers can be found also in **a** of this graph.
